# Supplementary material for: Comparative multiomics analysis of cell physiological state after culture in a basket bioreactor
Source: Sci Rep. 2022 Nov 23;12:20161. doi: 10.1038/s41598-022-24687-4 (PMC9686226; doi:10.1038/s41598-022-24687-4)
Supplement: Supplementary file 1 — Supplementary Information 1. [file 41598_2022_24687_MOESM1_ESM.zip › raw data/Transcriptomic raw data/7.AS/2.dasplot/TE1vsTC1/A5SS/19_EPB41_NW_023666033.1_12776278_12776313_+@NW_023666033.1_12776278_12776328_+@NW_023666033.1_12806779_12806907_+.pdf]

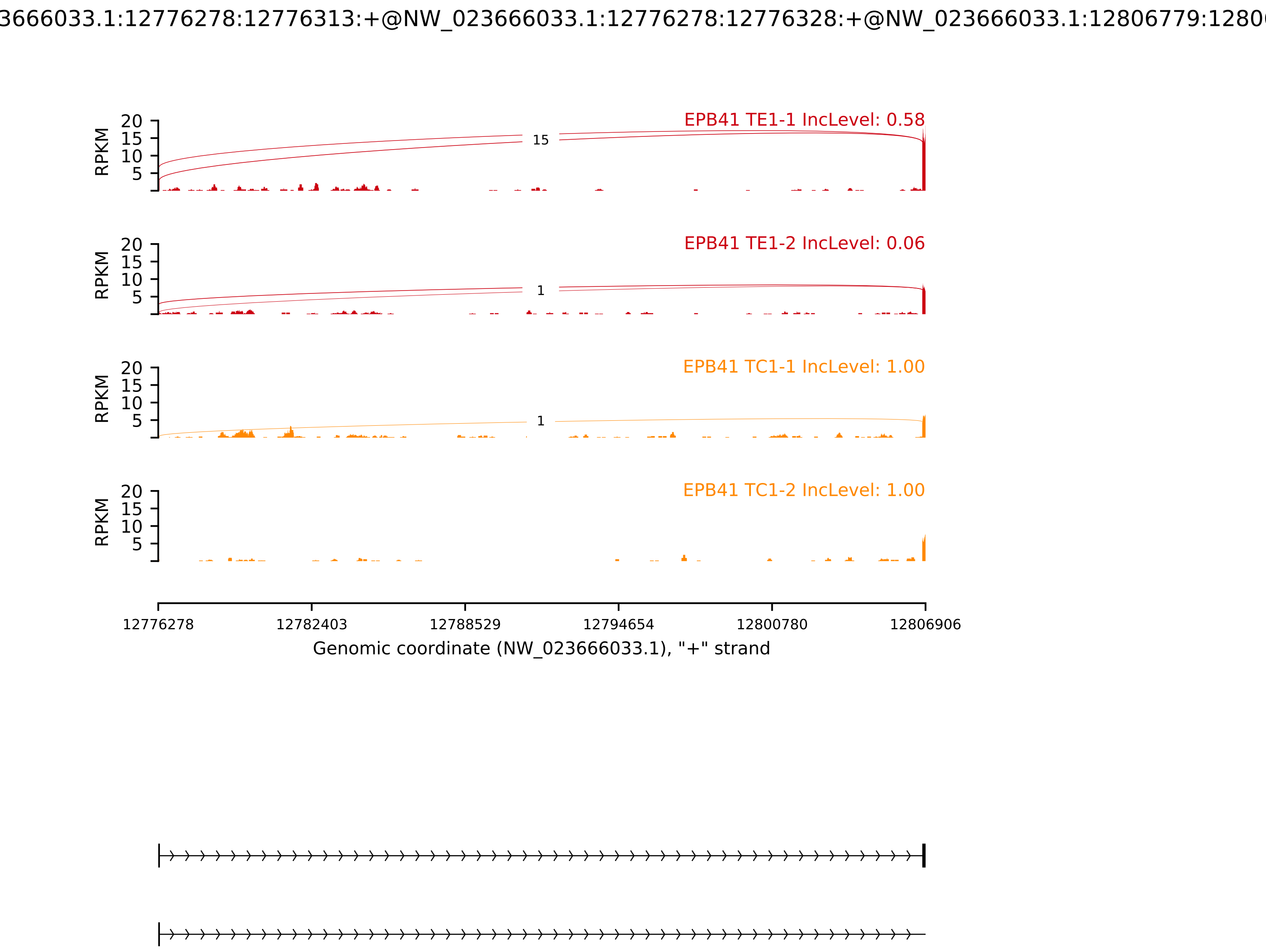
3666033.1:12776278:12776313:+@NW_023666033.1:12776278:12776328:+@NW_023666033.1:12806779:1280
EPB41 TE1-1 IncLevel: 0.58
15
EPB41 TE1-2 IncLevel: 0.06
1
EPB41 TC1-1 IncLevel: 1.00
1
EPB41 TC1-2 IncLevel: 1.00
RPKM
20
15
10
5
12776278
12782403
12788529
12794654
12800780
12806906
Genomic coordinate (NW_023666033.1), "+" strand
